# Supplementary material for: A Marine λ-Oligocarrageenan Inhibits Migratory and Invasive Ability of MDA-MB-231 Human Breast Cancer Cells through Actions on Heparanase Metabolism and MMP-14/MMP-2 Axis
Source: Mar Drugs. 2021 Sep 28;19(10):546. doi: 10.3390/md19100546 (PMC8539239; doi:10.3390/md19100546)
Supplement: Supplementary file 1 [file marinedrugs-19-00546-s001.zip › marinedrugs-1329117-supplementary.pdf]

Supplementary:

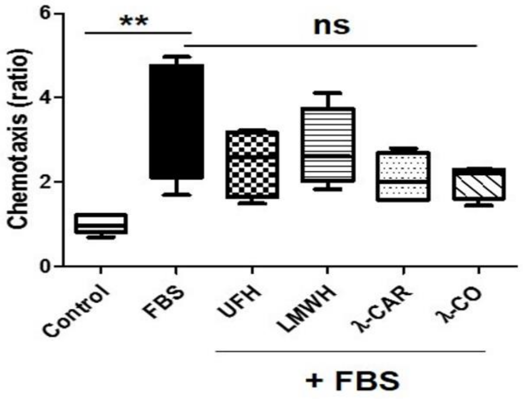


**Figure S1.** Short-term effects of the candidates on cell chemotaxis. Quantification of MDA cell migration through Boyden's chamber filter upon vehicle (control) or candidate's treatment at 100 μg/mL for 24 h. 10% FBS was used in the lower chamber as chemoattractant. Values are the mean ± SEM of three independent experiments. *p:* ** < 0.01; ns: non-significant. ANOVA followed by post hoc Fisher's LSD test.

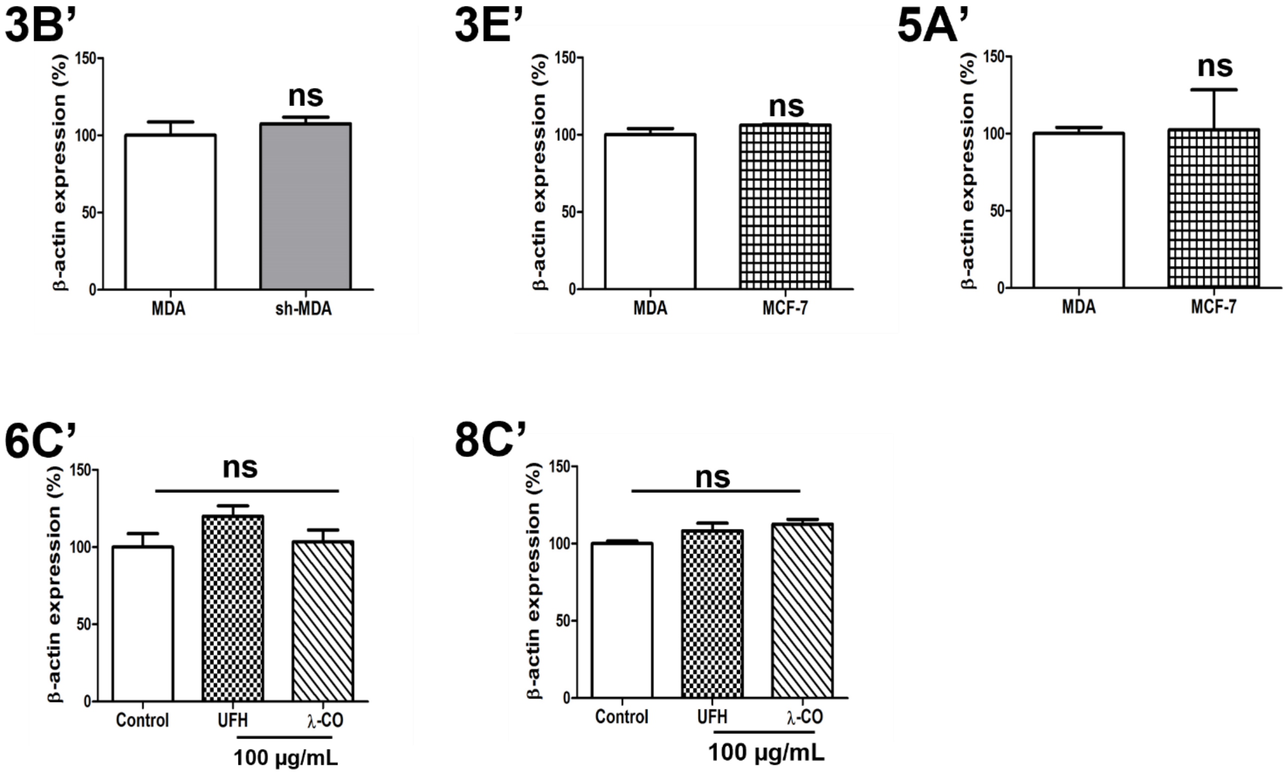


**Figure S2.** Quantification of β-actin levels. WB analyses of β-actin levels on MDA, sh-MDA and MCF-7 cells under control conditions or after 24 h treatment with UFH and λ-CO at 100 μg/mL or after HPSE downregulation for figures 3B, 3E, 5A, 6C, and 8C. Data are shown as the mean ± SEM of three independent experiments. Values are the mean ± SEM of three independent experiments. Student t test for 3B', 3E' and 5A' and ANOVA followed by post hoc Fisher's LSD for 6C' and 8C'. ns: non-significant

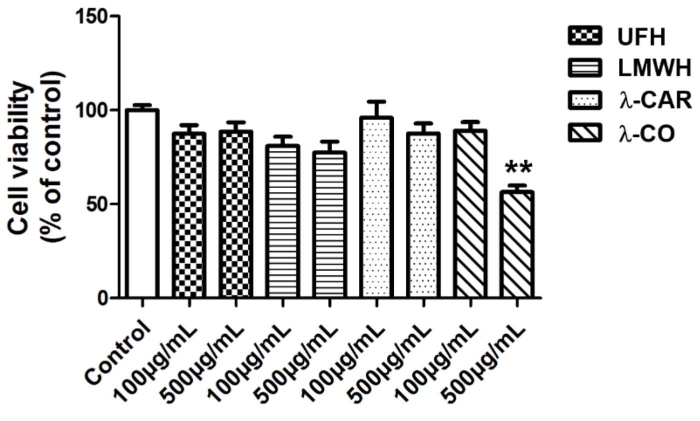


**Figure S3.** Effects of higher concentration of the four compounds on cell viability. Unfractionated Heparin (UFH), Low Molecular Weight Heparin (LMWH), native λ-Carrageenan (λ-CAR) and depolymerized λ-CAR (λ-CO) on MCF-7 cell viability and proliferation rate. MTT assay was used to analyse cell viability of cells treated with vehicle (control) or compounds at 100 and 500 μg/mL for 24 h. Values are the mean ± SEM of three independent experiments. ANOVA followed by post hoc Fisher's LSD test. ** $p < 0.01$.
